# SEMA4D Knockdown Attenuates *β*-Catenin-Dependent Tumor Progression in Colorectal Cancer

**DOI:** 10.1155/2021/8507373

**Published:** 2021-07-21

**Authors:** Mahsa Rezaeepoor, Golnaz Rashidi, Mona Pourjafar, Chiman Mohammadi, Ghasem Solgi, Rezvan Najafi

**Affiliations:** ^1^Department of Immunology, Faculty of Medicine, Hamadan University of Medical Sciences, Hamadan, Iran; ^2^Research Center for Molecular Medicine, Hamadan University of Medical Sciences, Hamadan, Iran

## Abstract

Semaphorin 4D (SEMA4D), a protein originally demonstrated to regulate the immune system and axonal growth cone collapse in the developing central nervous system, is overexpressed in various human malignancies, including colorectal cancer (CRC). This investigation was undertaken to examine the effects of SEMA4D silencing on the biological properties of the CRC cell line. SW48 cells were transfected with a siRNA-targeting SEMA4D. The mRNA expression of underlying pro- and antiapoptotic proteins including Bax, Bcl-2, P53, and caspase-3, cancer stem cell (CSC) markers, epithelial-mesenchymal transition (EMT) markers, MMP-2, and MMP-9 was examined using qRT-PCR. Further, the protein expression of E-cadherin and *β*-catenin was confirmed by Western blot. SW48 cell migration and MMP activity were detected using scratch and zymography analysis, respectively. Finally, the apoptosis rate was assessed via the flowcytometry test. SEMA4D knock-down was associated with a considerable suppression of in vitro cell viability, EMT-related genes, CSC markers, *β*-catenin signaling pathway, sphere-forming, cell migration, and MMP-2 activity as well as induction of apoptosis. This study identifies the inhibitory effects of SEMA4D gene silencing on tumor progression. Thereby, this might conclude a possible alternative to cancer therapy by targeting several prominent pathways involved in cancer through SEMA4D suppression.

## 1. Introduction

Colorectal cancer (CRC) is the third most prevalent malignancy as well as the second major cause of cancer-related mortality all over the world [1]. Several therapeutic modalities have been proposed for CRC based on pathological characteristics of the tumor such as surgery, radiotherapy, and chemotherapy [2]. Recurrence, metastasis, and resistance to treatment have caused problems in the therapy of CRC [3]. Tumors consist of several kinds of cancer cells contributing to the heterogeneity of the tumor. Several investigations have indicated that a number of malignant cells like colon cancer cells acquire the features of cancer stem cells (CSCs) via epithelial-mesenchymal transition (EMT). CSCs play a crucial role in the initiation and progression of cancer [4, 5]. Brabletz et al. have proved the presence of two stem cell subpopulations in colon cancer. The first was able to initiate the tumor and the second could spread the tumor-forming metastasis. Hence, the CSC theory has been presented as a novel perspective to explore tumor initiation, recurrence, and metastasis [6].

Epithelial-mesenchymal transition (EMT) is a process by which cancer cells attain an invasive phenotype leading to metastasis. During the progression of tumor, EMT contributes remarkably to the malignant features of tumors, including local invasion and distant metastasis. EMT has recently been recognized as a key phenomenon that tightly adjusts the CSCs [7]. During the EMT process, the transformed epithelial cells acquire mesenchymal traits that convert them to metastatic cells. A hallmark of metastatic cells is the lost expression of epithelial markers such as E-cadherin as well as induction of mesenchymal markers including N-cadherin, vimentin, and Zeb1 [8]. Several signaling pathways, including hepatocyte growth factor (HGF), epidermal growth factor (EGF), transforming growth factor beta (TGF-*β*), Notch, and Wnt/*β*-catenin, regulate EMT, and the activation of Wnt/*β*-catenin pathway is commonly seen in many malignant tumors [9, 10].

Wnt is a vital signaling pathway associated with self-renewal of tissues and homeostasis. *β*-Catenin is a prominent mediator of Wnt signaling pathway, leading to signal transmission from the cytoplasm through the nucleus [11]. Activation of WNT/*β*-catenin signaling can result in *β*-catenin accumulation in the nucleus, which is accompanied with a poor prognosis among CRC patients [12]. Recent findings have evidently suggested the critical effect of WNT/*β*-catenin dysregulation on the induction of EMT process as well as sustained CSC expansion in the pathogenesis of CRC [11, 13].

The semaphorins are glycoproteins discovered in 1990s and initially described as axon guidance factors, which have later been implicated in the regulation of immune responses, angiogenesis, as well as pathogenic processes, including tumor inhibition and progression [14]. Semaphorin 4D (SEMA4D) was originally described in immune cells as CD100 antigen and was the first semaphorin family member found to possess immunoregulatory activities. SEMA4D is synthesized as a transmembrane molecule, the soluble extracellular portion of which can be shed following metalloprotease-mediated cleavage. Notably, SEMA4D has three known receptors, namely, the high, intermediate, and low affinity PlexinB1, PlexinB2, and CD72, respectively, which are mainly expressed by immune cells [15]. Plexin-B1 is expressed on vascular endothelial cells (VECs) at high levels, inducing angiogenesis by an independent mechanism apart from other angiogenic promoters such as vascular endothelial growth factor (VEGF-a), basic fibroblast growth factor (bFGF), and HGF [16]. Upregulation of Plexin-B1 is demonstrated on breast, pancreatic, and colorectal carcinoma tissues [17–19]. As an immune semaphorin, SEMA4D is able to regulate tumor microenvironment by differentiating monocytes toward tumor-supportive macrophage phenotype, namely, M2 macrophages with high expression of CD163 [20]. Moreover, SEMA4D is elevated in a variety of tumor tissues relative to normal tissue cells, including breast, cervical, epithelial ovarian, and prostate cancers as well as CRC [18, 21–24]. It has been reported that the coexpression of SEMA4D and PlexinB1 is a risk factor for CRC relapse and that it may be an effective prognostic biomarker for recurrence of CRC [17]. Targeted therapy is an important emerging area for cancer treatment. Based on clinical applicability of semaphorins and their receptors as well as their prominent roles especially in the tumor progression, semaphorins have turned into compelling therapeutic targets, then we followed the methods of our previous study [25].

The goal of the present study was to determine the changes of tumor progression following the inhibition of SEMA4D expression using siRNA. The findings indicated that targeting SEMA4D for inhibition might serve as a therapeutics solution for the suppression of invasion, migration, and viability of SW48 cell line, which is related to CRC.

## 2. Materials and Methods

### 2.1. Cell Culture

As a model of colon cancer cells, the SW48 human colorectal cancer cell line, purchased from the National Cell Bank of Pasteur Institute (Tehran, Iran), was used. The cells were cultured in Dulbecco's modified Eagle's medium supplemented with 10% heat-inactivated fetal bovine serum (FBS; Gibco, Waltham, MA) and 1% penicillin or streptomycin (Gibco), then were incubated in a humidified incubator with a temperature of 37°C and 5% CO2 for 72 h.

### 2.2. Cell Transfection

SEMA4D siRNA was purchased from Bioneer (South Korea) and cells at about 60–70% confluence were transfected with siRNA (30 nmol) using HiPerfect Transfection Reagent (Qiagen, USA). SW48 cells were also transfected with scrambled siRNA as it does not target any known mammalian gene. Briefly, 10^5^ cells were seeded (per well) in a 12-well plate. For each transfection sample, a complex was prepared: the siRNA was diluted in 100 *μ*l of Opti-MEM medium (Gibco) and 9 *μ*l of HiPerfect. The complex was blended gently and was incubated 15 min at room temperature. Before adding the complex, the culture medium was renewed (without FBS and Pen/strep). Then, transfection complex was added to each well, containing cells and medium, and mixed softly by rocking the plate back and forth. Eventually, the plate was incubated at 37°C in a humidified incubator with 5% CO2 for 72 h.

### 2.3. Cell Viability Assay

To evaluate cell viability, the colorimetric MTT assay was used. Cells were seeded in 96-well plates at a density of 8000 cells in 50 *μ*l per well, and then, cells were treated with final concentrations of siRNA (30 nmol). After incubation for 24 hours, 3-(4, 5-Dimethylthiazol-2-yl)-2, 5-diphenyltetrazolium bromide (Sigma Aldrich, St Louis, MO, USA) was added and incubated for 4 hours at 37°C. The medium was discarded, and then, 100 *μ*l DMSO (Sigma-Aldrich) was added. The optical density of solubilized Formazan was measured at 570 nm using an automatic microplate reader.

### 2.4. RNA Isolation and Quantitative Reverse-Transcription Polymerase Chain Reaction (RT-qPCR)

Total RNA was isolated from the SW48 cell line using RNX-Plus Kit (Cinnagen, Tehran, Iran), 72 h after transfection, as recommended by the manufacturer. Purity and concentration of RNA were evaluated with NanoDrop, and total RNA was turned to cDNA with reverse transcriptase using a first-strand cDNA synthesis kit (Takara, Japan). Real-time PCR assay was conducted in triplicate using RealQ plus 2x master (Ampliqon, Denmark). The standard PCR conditions were as follows: 95°C for 15 min incubation, followed by 40 amplification cycles of denaturation at 95°C for 30 s, annealing at 61°C for 30 s and extension 72°C for 30 s using a light cycler instrument (Roche 96 system, Germany). Primer sequences used for this study are listed in Table 1. Data were analyzed by normalizing with GAPDH as an internal control, and the relative expression of genes was plotted using the 2^−ΔΔCt^ method.

### 2.5. Western Blot Analysis

Total protein was isolated from harvested SW48 cells after 72 h using 0.2 ml of radioimmuno precipitation assay (RIPA) buffer (Santa Cruz, USA), and concentration was measured by the Bradford protein assay. 100 *μ*g proteins with equal volumes were separated on 10% sodium dodecyl sulfate-polyacrylamide gel electrophoresis (SDS-PAGE) and then transferred from the gel onto a nitrocellulose membrane. After blocking, first antibodies including rabbit monoclonal anti-E-cadherin (ab40772, 1 : 7,000), rabbit monoclonal anti-*β*-catenin (ab32572, 1 : 5000), and rabbit anti-beta-actin (ab8227, 1 : 5,000) were incubated with the membrane at 4°C overnight. The secondary antibody (anti-rabbit antibody conjugated to horseradish peroxidase) was used, and bands were visualized using enhanced chemiluminescence detection kit. To quantify the intensity of bands, the ImageJ software was used.

### 2.6. Zymography

To evaluate the MMP-2/-9 enzymatic activities, zymography was performed. First, the supernatants of SW48 cell line (controls and transfected cells) were collected. Then, defined amounts of proteins were loaded in 10% (*w*/*v*) polyacrylamide gels containing 1 mg gelatin/ml. Electrophoresis was run at 120 V for 2 h. To renature the gel, it was immersed in 2.5% Triton X-100 buffer for 2 h and then incubated in developing buffer for 72 h at 37°C (50 mM Tris–HCl, 200 mM NaCl, 5 mM CaCl2, and 0.01% NaN3, pH 7.5). Finally, the gel was stained with 0.1% Coomassie brilliant blue G-250 in a 40% methanol and 10% acetic acid solution and then destained in 40% methanol, 10% acetic acid, and deionized water. Gel was scanned, and vivid bands were analyzed by NIH ImageJ software.

### 2.7. Flow Cytometry-Based Apoptosis Analysis

Apoptosis of the cells was measured according to the manufacturer's instructions, using an Annexin-V-FITC/Propidium Iodide (PI) kit (MabTag, Germany). Briefly, SW48 cell lines were collected after transfection. After washing and centrifuging at 300 g for 5 min at 4°C, the cell pellet was resuspended in cold binding buffer. The Annexin V-FITC and PI solutions were added to the cell suspension and gently mixed. Following 15 min of incubation time, apoptosis and cell death were analyzed using Attune NxT acoustic focusing cytometer (Life technology, USA) and FlowJo software 10.

### 2.8. Colony-Forming Assay

To determine cell growth and survival, colony-formation assay was conducted [26]. 400 cells (per well) of transfected SW48 and control were seeded on a 6-well plate and allowed to grow for 10 days. The cell colonies were then stained with 5% crystal violet for 45 min. After that, they were washed twice with PBS and distilled water and counted. To evaluate efficiency, the ratios of the visible colonies from all seeded cells were calculated. The survival fraction was clarified by the percentage of transfected cells compared to the control.

### 2.9. Sphere Formation Assay

After 72 h of transfection, SW48 cells were seeded in a 6-well low attachment plate at a density of 30,000 cells per well with 20 ng/ml of epidermal growth factor (EGF) and 20 ng/ml of basic fibroblast growth factor (b-FGF). After 7 days of incubation at 37°C, the number of formed spheroids >50 *μ*m in diameter was counted using ImageJ software [27, 28].

### 2.10. Wound Healing Assay

Briefly, 10^5^ SW48 were seeded into 12-well plates. 72 h after transfection, an incision was made on the cell layers with a sterile pipette tip (100 *μ*l). To examine the cell migration over the incision, an inverted microscope was used after 24-48 h, and finally, distances in the scratch area were analyzed by the NIH Image J software.

### 2.11. Statistical Analysis

Evaluation of data analysis among different groups was performed by PRISM software using unpaired *T*-tests. Data were reported as the mean ± SD and compared by analysis of variance; *P* < 0.05 was considered statistically significant.

## 3. Results

### 3.1. siRNA SEMA4D Suppressed SEMA4D Expression and Viability of SW48 Cells

**Figure 1:**
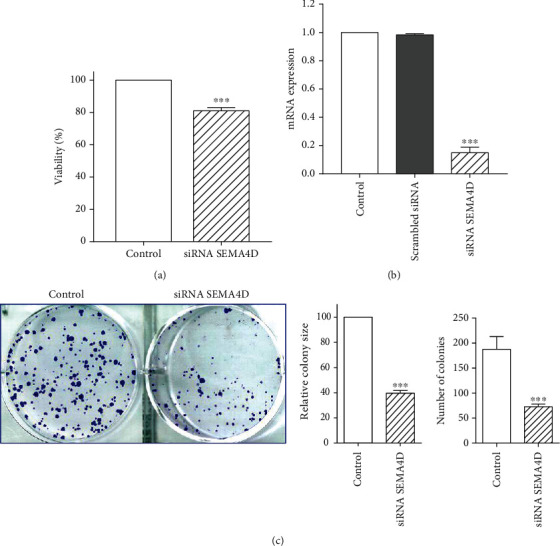
Effect of siRNA SEMA4D on SW48 cells viability. (a) MTT analysis of SW48 cells in the presence and absence of siRNA SEMA4D. (b) Cells were transfected with SEMA4D siRNA and scrambled siRNA and untransfected as control. RT-qPCR analysis revealed the downregulation of SEMA4D in siRNA SEMA4D transfected cells. (c) The effect of siRNA SEMA4D on the proliferation of SW48 cells was analyzed by colony formation assay. The findings are expressed as mean ± standard deviation (SD) of three independent experiments. ^∗∗∗^*P* < 0.001 compared with control.

SEMA4D siRNA was transfected into cells and the downregulation of SEMA4D was evaluated by RT-qPCR. As reported in an our previous study, SEMA4D siRNA caused markedly inhibition in the expression of SEMA4D in SW48 cells [25]. As it is also shown in Figure 1(b), there was no remarkable difference between the untransfected control and scrambled siRNA transfected cells for SEMA4D expression which confirmed the specific silencing effect of siRNA SEMA4D. Then, we performed MTT assay to investigate the influence of SEMA4D on cell viability and metabolic activity. As shown in Figure 1(a), the ratio of viable cells was decreased in the cells transfected by siRNA SEMA4D in comparison with control.

Since the ability to proliferate and generate colonies is a key feature of cancer cells, the colony forming capacity of transfected SW48 cells was investigated in this study. siRNA SEMA4D remarkably decreased the number and size of colonies relative to the control group (Figure 1(c)).

### 3.2. Downregulation of SEMA4D Induces Apoptosis in SW48 Cells

**Figure 2:**
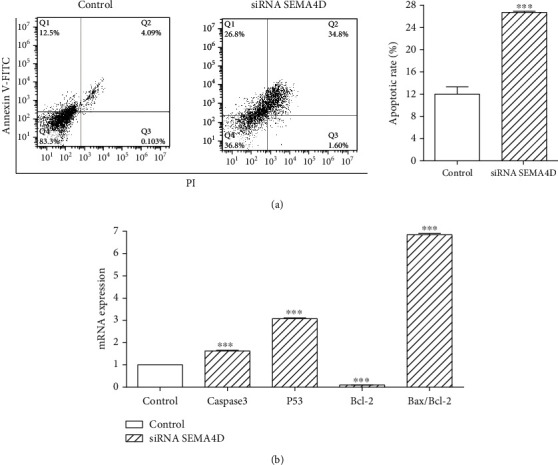
Effect of siRNA SEMA4D on SW48 cells apoptosis. (a) Induction of apoptosis was measured by flow cytometry analysis after 72 h in the presence and absence of siRNA SEMA4D. (b) mRNA levels of caspase 3, P53, Bcl2, and Bax were analyzed by qRT-PCR in transfected and untransfected SW48 cells. GAPDH was employed as an internal control. The data are expressed as mean ± standard deviation, and each experiment was repeated three times. ^∗∗∗^*P* < 0.001 vs. control group.

The effect of SEMA4D knockdown on apoptosis was assessed using Annexin V/PI assay. Flow cytometric analysis demonstrated that SEMA4D silencing increases the percentage of apoptotic cells compared to untransfected cells (Figure 2(a)). To check the effect of SEMA4D on the induction of apoptosis, we evaluated the expression of apoptosis-related genes. mRNA expression levels of Caspase-3, P53, Bcl2, and Bax were thus analyzed. After SEMA4D depletion, mRNA expression levels of Caspase-3, P53, as well as Bax/Bcl2 ratio significantly increased, whereas there was a marked decrease in mRNA expression of Bcl-2 gene in SW48 cells transfected with siRNA SEMA4D in comparison with control group (Figure 2(b)).

### 3.3. Knockdown of SEMA4D Suppressed CSCs and EMT Markers via Downregulation of *β*-Catenin

**Figure 3:**
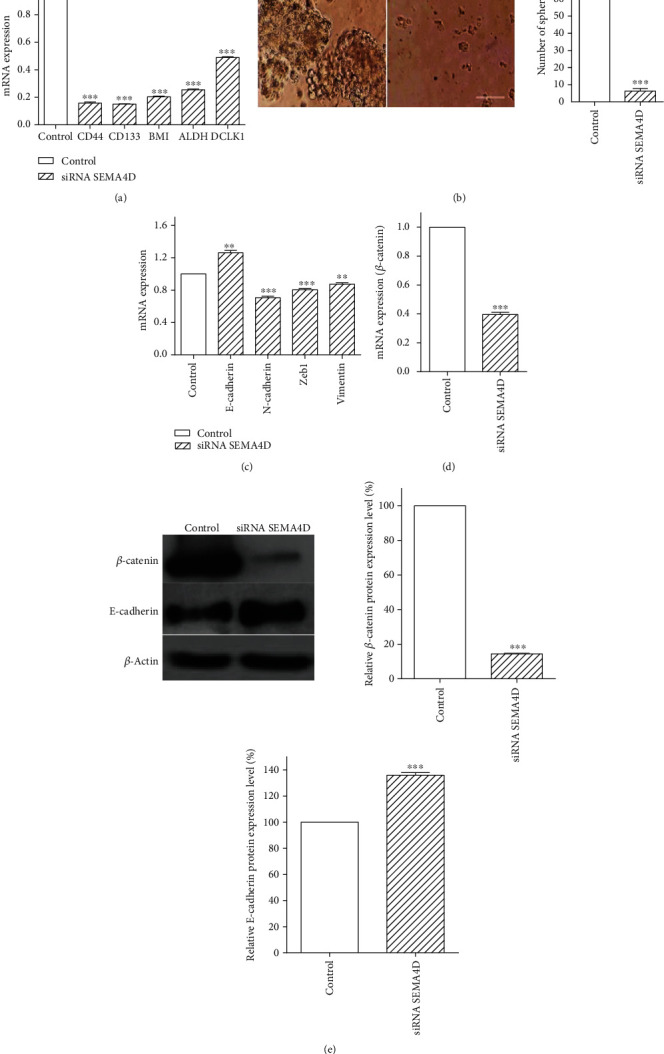
Effect of SEMA4D silencing on EMT- and CSC-related marker expression. (a) The mRNA expression levels of CSC-related genes were examined by RT-qPCR. (b) Representative images from sphere-forming assay. (c) RT–qPCR analysis of EMT-related markers. (d) Transcript abundance of *β*-catenin was analyzed by RT-qPCR. (e) *β*-catenin and E-cadherin protein expressions were determined by Western blot, and bar graph shows semiquantified densitometry from Western blot analysis. The data are expressed as mean ± standard deviation, and each experiment was repeated three times. ^∗∗∗^*P* < 0.001 vs. control group.

As fundamental physiological processes, CSCs and EMT play critical roles in chemoresistance, invasion, tumor recurrence, and metastasis of tumor cells. To evaluate the effect of SEMA4D suppression on CSCs and EMT markers, SW48 cells were transfected with siRNA SEMA4D for 72 h, and their mRNA expression was checked using RT-qPCR. Our findings revealed that transcript expression of CSC markers, including CD44, CD133, BMI1, ALDH1, and DCLK1, was significantly lower in transfected SW48 cells relative to untransfected cells (Figure 3(a)). A crucial feature of CSCs is their capacity to form spheres when growing in serum-free medium within nonadherent plates. To confirm the inhibition of CSC markers by siRNA SEMA4D, sphere formation assay was done to examine the ability of CSC self-renewal in SW48 cells. The findings indicated that the size and number of spheres in transfected SW48 cells was much lower after 7 days compared to control cells (Figure 3(b)).

Moreover, it was demonstrated that SEMA4D knockdown significantly increased the expression of E-cadherin as an epithelial marker, while mesenchymal markers such as N-cadherin, Zeb1, and vimentin were remarkably reduced in comparison with control group (Figure 3(c)).

In addition, mRNA and protein expression levels of *β*-catenin as an oncogenic transcription factor were, respectively, analyzed by RT-qPCR and Western blot to illustrate the mechanism underlying the decrease in cancer stemness and EMT markers through silencing of SEMA4D. Our data revealed that mRNA and protein expression of *β*-catenin were strongly suppressed upon SEMA4D silencing (Figures 3(d) and 3(e)). In addition, Western blot analysis was performed to confirm the gene expression results of E-cadherin. The findings indicated a considerable increase of E-cadherin protein expression in the presence of siRNA SEMA4D (Figure 3(e)).

### 3.4. SEMA4D Suppression Decreases SW48 Cells Migration

**Figure 4:**
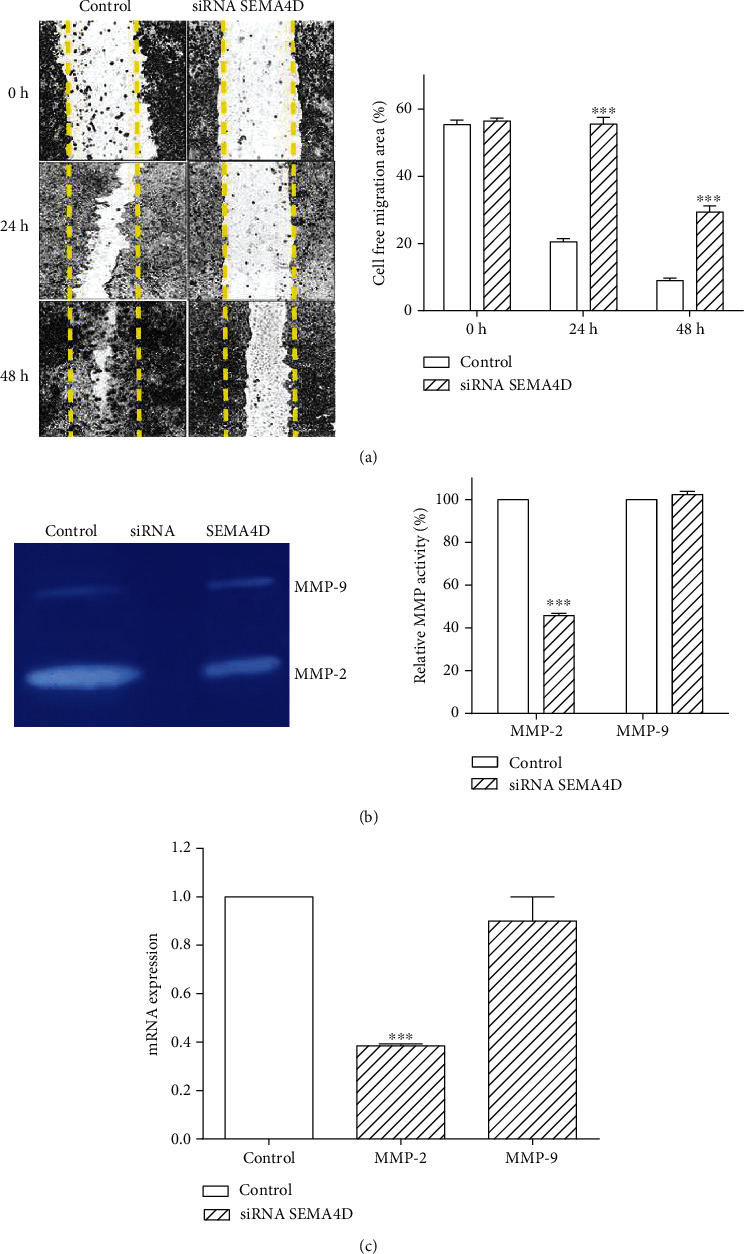
Effect of SEMA4D silencing on cell migration. (a) Representative image of SW48 cell migration in the absence and presence of siRNA SEMA4D at 0 h, 24 h, and 48 h. The scratched areas were measured in three random fields in each group. (b) The effect of siRNA SEMA4D on MMP-2 and MMP-9 activity, which was evaluated by gelatin zymography. Gelatinolytic activity of MMP-2 and MMP-9 is revealed in polyacrylamide gel by white banding. (c) The MMP-2 and MMP-9 gene expression was analyzed by real-time PCR. Data are represented as mean ± standard deviation^∗∗∗^*P* < 0.001 vs. control group.

Scratch test was performed to detect the impact of SEMA4D silencing on migration ability of SW48 cells. Our findings exhibited that following transfection by siRNA, the scratches were wider than those of untransfected cells due to decreased number of cells migrating from the edge of the wound (Figure 4(a)). In addition, zymography was employed to measure hydrolytic activity of matrix metalloproteinase-2/-9 (MMP-2/-9). The zymographic activity of MMP-2 was markedly decreased in transfected SW48 cells compared with untransfected cells. There was no considerable change in the activity of MMP-9 (Figure 4(b)). Real-time PCR analysis revealed that the expression of MMP-2 was remarkably diminished relative to control, and again, there was no significant difference in MMP-9 mRNA expression (Figure 4(c)).

## 4. Discussion

CRC remains one of the leading cause of human malignancies worldwide [29]. Although pioneering treatment strategies have been made, its morbidity and mortality are still substantial. Therefore, alternative strategies might be beneficial and effective. SEMA4D plays key roles in various mechanisms involved in cancer including tumor angiogenesis, cancer progression, metastasis, and also, tumor microenvironment regulation [30–32]. Notably, SEMA4D is overexpressed in various malignancies compared with normal tissue cells, such as prostate cancer [33], pancreatic cancer [18], cervical cancer [23], and particularly CRC [17, 24]. Detection of SEMA4D is correlated with a poor prognosis in CRC patients and can be used for predicting disease recurrence in CRC patients [17, 34].

It has been reported that lymphocytes infiltrated in the tumor stroma of pancreatic and colon cancer are responsible for SEMA4D expression [17, 18]. However, several investigations reported that tumor-associated macrophages (TAMs) considerably induced expression of SEMA4D in colon cells, and this overexpression is remarkably associated with lymphatic metastasis and specific histological types [24, 35]. A study conducted by Evans et al. illustrated an antitumor activity of SEMA4D blocking through antibody on the immune system [36]. Moreover, dihydromyricetin (DMY) exerted its antitumor effects through induction of the oxidative stress and inflammation in colon cancer cells following regulation of the SEMA4D expression [37].

While the expression abundance of SEMA4D has been investigated in the colorectal tissues, the correlation between silencing of SEMA4D expression and colon cell responses remains relatively unclear. Accordingly, the comprehension of new cellular and molecular mechanisms involved in tumor progression is pivotal for the development of novel therapeutic approaches. In our former study, it was showed that siRNA-mediated SEMA4D gene silencing can increase chemosensitivity to 5-FU in CRC through several oncogenic pathways particularly by promoting apoptosis [25], but in this study, we aimed to investigate whether SEMA4D inhibition can directly be effective in tumor suppression.

Hence, the purpose of this study was to demonstrate the changes in tumor progression mechanisms such as apoptosis, migration, EMT process, and *β*-catenin pathway followed by the silencing of SEMA4D expression using siRNA. This is, to our knowledge, the first study to investigate the relationship between knocking down of SEMA4D expression and *β*-catenin pathway-dependent EMT and CSCs in CRC.

In the present study, after the SEMA4D knockdown, the percentage of viable tumor cells decreased, and the rate of apoptosis increased. Moreover, SEMA4D silencing inhibited SW48 cell migration, the CSC markers, EMT process, *β*-catenin signaling pathway, colony formation, sphere formation, and MMP-2 activities.

CSCs are responsible for tumor initiation, tumor progression, and therapeutic resistance. Conventional CRC therapies are challengingly failed to target CSCs because of their extreme persistence to the treatment; consequently, they may be causative of tumor recurrence and metastasis [38]. The present observations support considerable downregulation of CSC markers such as CD44, CD133, ALDH, DCLK1, and BMI1 as well as inhibition of sphere-forming assay as an in vitro assay for the determination and enrichment of CSCs. Interestingly, CSCs can participate in TAMs infiltration and polarization in tumor stroma [39]. Therefore, SEMA4D silencing not only might directly lead to a decrease of CSCs but also, it might indirectly contribute to the suppression of TAMs recruiting. Moreover, TAMs implicate tumor progression through EMT and angiogenesis. Subsequently, acquiring an EMT phenotype leads to cancer cell invasion and tumor metastasis.

Several lines of evidence have illustrated that CRC cells with an EMT phenotype are rich sources for CSCs, indicating a biological link between EMT and CSCs [38]. Here, after inhibition of SEMA4D, the expression levels of EMT markers including mesenchymal markers such as N-cadherin and vimentin as well as EMT transcription factor (Zeb1) were diminished compared with untreated cells; however, E-cadherin as an epithelial marker was elevated. E-cadherin plays an underlying role in the inhibition of invasion, and its regulation might result in repression of tumor progression (15).

Activation of EMT is induced through a prominent self-renewal pathway: WNT/*β*-catenin. It is demonstrated that overactivation of the WNT/*β*-catenin pathway could repress E-cadherin and promote EMT process and local invasion in the colorectal tumor [40, 41]. Our data revealed that the mRNA expression of *β*-catenin and its protein level downregulated following transfection of the siRNA SEMA4D. Therefore, *β*-catenin oncogene inhibition might be effective on enhanced E-cadherin and suppressed EMT and CSC markers. Indeed, it has been demonstrated that *β*-catenin suppression which is considered as a growth stimulus in a subset of colon cancer cell lines led to tumor regression [42, 43]. Furthermore, it has been reported that the depletion of E-cadherin contributes to induce EMT, eventually leading to migration [44].

Another critical factor for tumor progression is migration via the ECM that involves several types of molecules, including MMPs and chemokine [45]. MMPs are expressed in different stages of CRC and linked with survival and prognosis [46]. In this article, we concentrated on MMP-2 and MMP-9, due to their high expression reporting by various cancers investigations including CRC [46, 47].

SEMA4D knockdown significantly repressed cell migration ability and mRNA expression of MMP-2 as well as its enzymatic activity, whereas the mRNA expression and enzymatic activity of MMP-9 stayed constant after transfection of siRNA SEMA4D. It is revealed that the cancer cells could promote invasion in an MMP2-dependent manner following E-cadherin knockdown [48] As reported by Yan Wang et al., SEMA4D expression has been reported to be increased in esophagus squamous cell carcinoma (ESCC), and consistent with our results, SEMA4D knockdown could repress cell proliferation and migration in ESCC [49].

Several studies in the course of recent years have indicated the significant role of apoptosis in various malignancies and its promising role in anticancer therapy [50]. Defect in apoptosis is a hallmark of human cancer and leads to an aggressive tumor phenotype correlating with drug resistance and failure of treatments including chemotherapy, radiation, and targeted therapies [51].

Apoptotic signaling pathways which are crucial for tumor survival deregulated in CRC, and this dysregulation mainly correlated with overexpression of Bcl-2 family proteins [52]. Evasion of apoptosis and the disproportionateness between proapoptotic and antiapoptotic protein levels are critical factors in the pathogenesis of cancer [53]. In addition, the apoptosis evasion of CSCs is considered as a prominent element for the failure of existing therapies to abolish tumors [52].

As predicted, our results demonstrate that SEMA4D knockdown markedly accelerated the rate of apoptotic cells compared with nontransfected cells. It was also associated with the reduced expression of Bcl-2 and the elevated expression of P53, caspase-3, and Bax/Bcl-2 ratio. Management of the apoptotic pathways, including deactivation of antiapoptotic pathways and stimulation of proapoptotic markers to eliminate tumor cells, demonstrates significant potentials. In line with our findings, it has been indicated that SEMA4D silencing elevated the apoptosis rate of KYSE-150 and TE-10 cells [49]. In addition, the upregulation of SEMA4D in HP45 cells could suppress the apoptosis, and SEMA4D targeting by miR-125b could negatively regulate it and augment the apoptosis rate in avian leucosis virus-transformed cells [54]. As reported by Liu et al., it has been revealed that targeting of SEMA4D by miR-214 could prevent cell proliferation and induce apoptosis in ovarian cancer cells [55]. The findings from the present study indicate that SEMA4D knockdown by siRNA in human colorectal cancer cells suppressed various oncogenic pathways, including inhibition of *β*-catenin pathway-dependent EMT and CSCs, attenuating cell growth and invasion through diminution MMP-2 enzymatic activity and apoptosis augmentation. It is possible that SEMA4D silencing may prevent TAM infiltration and polarization in tumor stroma which is potentially required for SEMA4D production. Further investigation will be necessary to elucidate the mechanism of suppressive effect of SEMA4D silencing on the recruiting of TAMs. Ultimately, this data suggest that SEMA4D might be considered a substantial cornerstone for the development of CRC-specific therapeutics.

## Figures and Tables

**Table 1:** Specific primer sequences for qRT-PCR.

Gene	Sense strand	Antisense strand
SEMA4D	AATGTTTGACGACACTGATGGT	TCTTTGCTGGTGCTAGAGATG
MMP2	GGAGCATGGCGATGGATACC	TTCACACGGACCACTTGGC
MMP9	GGTGATTGACGACGCCTTTG	AACCGAGTTGGAACCACGA
Bax	CCGCCGTGGACACAGACT	TTGAAGTTGCCGTCAGAAAACA
Bcl-2	TGGAGAGTGCTGAAGATTGA	GTCTACTTCCTCTGTGATGTTGTAT
P53	TAACAGTTCTGCATGGGCGGC	AGGACAGGCACAAACACGCACC
CD44	AATGGTCGCTACAGCATCTC	GCCCTTCTATGAACCCATACC
CD133	GAGTCGGAAACTGGCAGATAG	AACGCCTTGTCCTTGGTAG
BMI1	CATCCACAGTTTCCTCACATTTC	GAAGTTGCTGATGACCCATTTAC
*β*-Catenin	CTTCACCTGACAGATCCAAGTC	CCTTCCATCCCTTCCTGTTTAG
E-cadherin	AGAACGCATTGCCACATACA	GAGGATGGTGTAAGCGATGG
N-cadherin	ATTCGGGTAATCCTCCCAAATC	CCCACAATCCTGTCCACATC
Vimentin	CATTGAGATTGCCACCTAC	CGTTGATAACCTGTCCATC
ZEB1	ATTCGGGTAATCCTCCCAAATC	TTTCACTGTCTTCATCCTCTTCCC
DCLK1	TTGCTCCAGATCGTTAGAAGG	CAGGAAGGTCTCATTGAACAC
GAPDH	GCCATCAATGACCCCTTCATT	TTGACGGTGCCATGGAATTT

## Data Availability

The data that support the findings of this study are available in the manuscript.

## Supplementary Materials

Supplementary Materials Uncropped Western blots corresponding to Figure 3(e).
