# Supplementary figures and images for: SEMA4D Knockdown Attenuates β-Catenin-Dependent Tumor Progression in Colorectal Cancer
Source: Biomed Res Int. 2021 Jul 21;2021:8507373. doi: 10.1155/2021/8507373 (PMC8321723; doi:10.1155/2021/8507373)

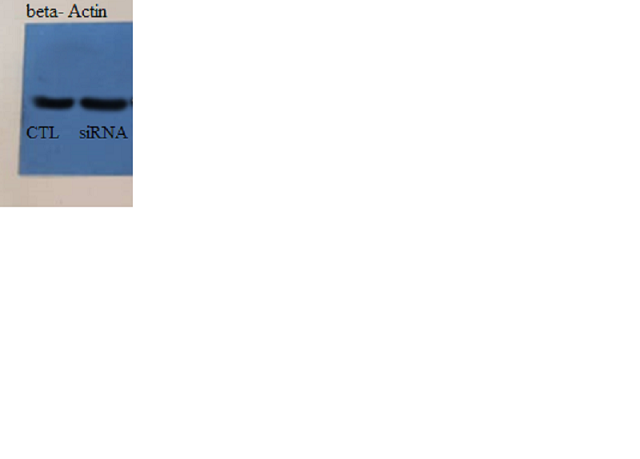

Supplement: Supplementary Materials — Uncropped Western blots corresponding to Figure 3(e). [file 8507373.f1.zip › beta actin (1).png]

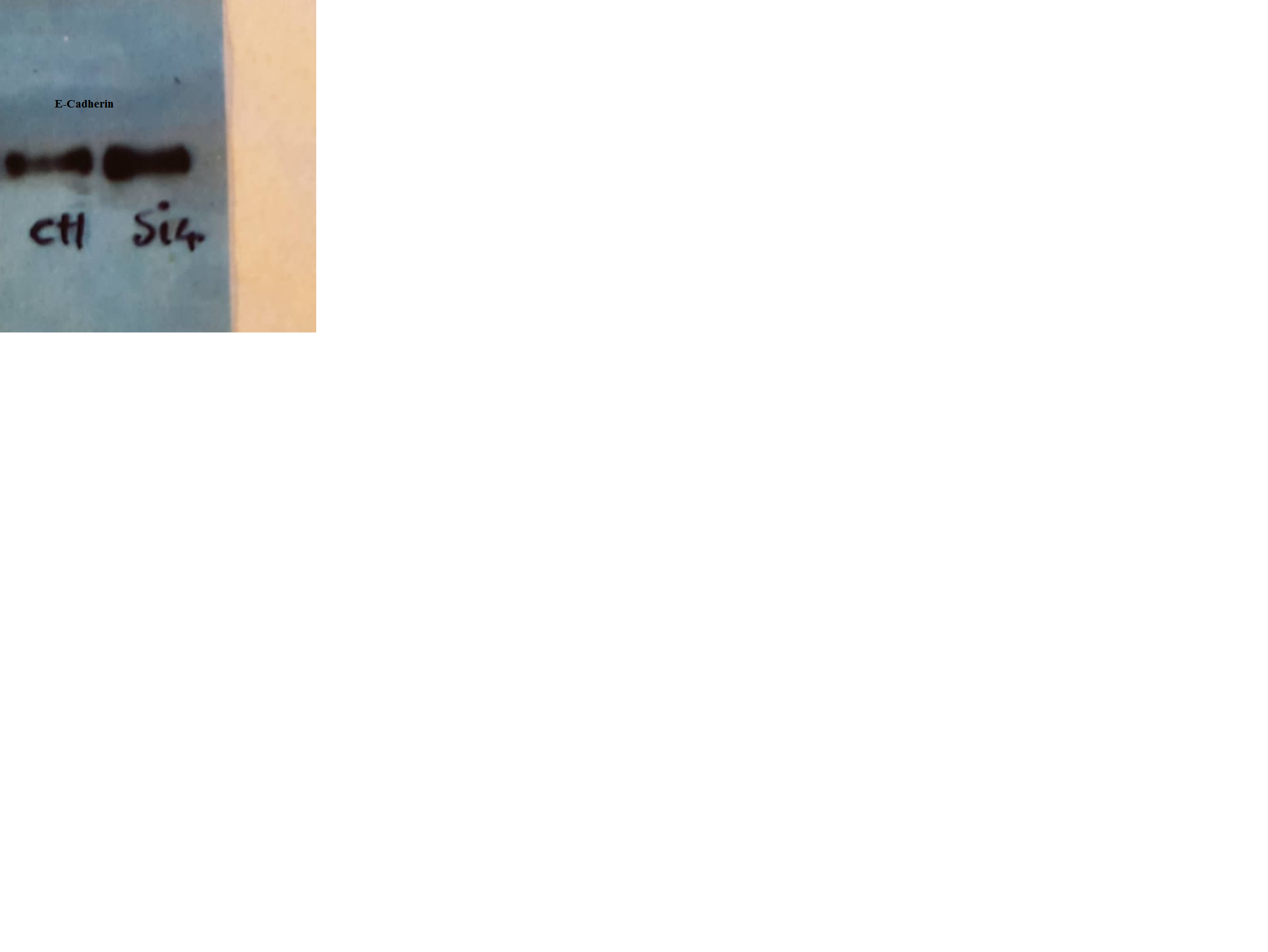

Supplement: Supplementary Materials — Uncropped Western blots corresponding to Figure 3(e). [file 8507373.f1.zip › e-cadherin (1).png]

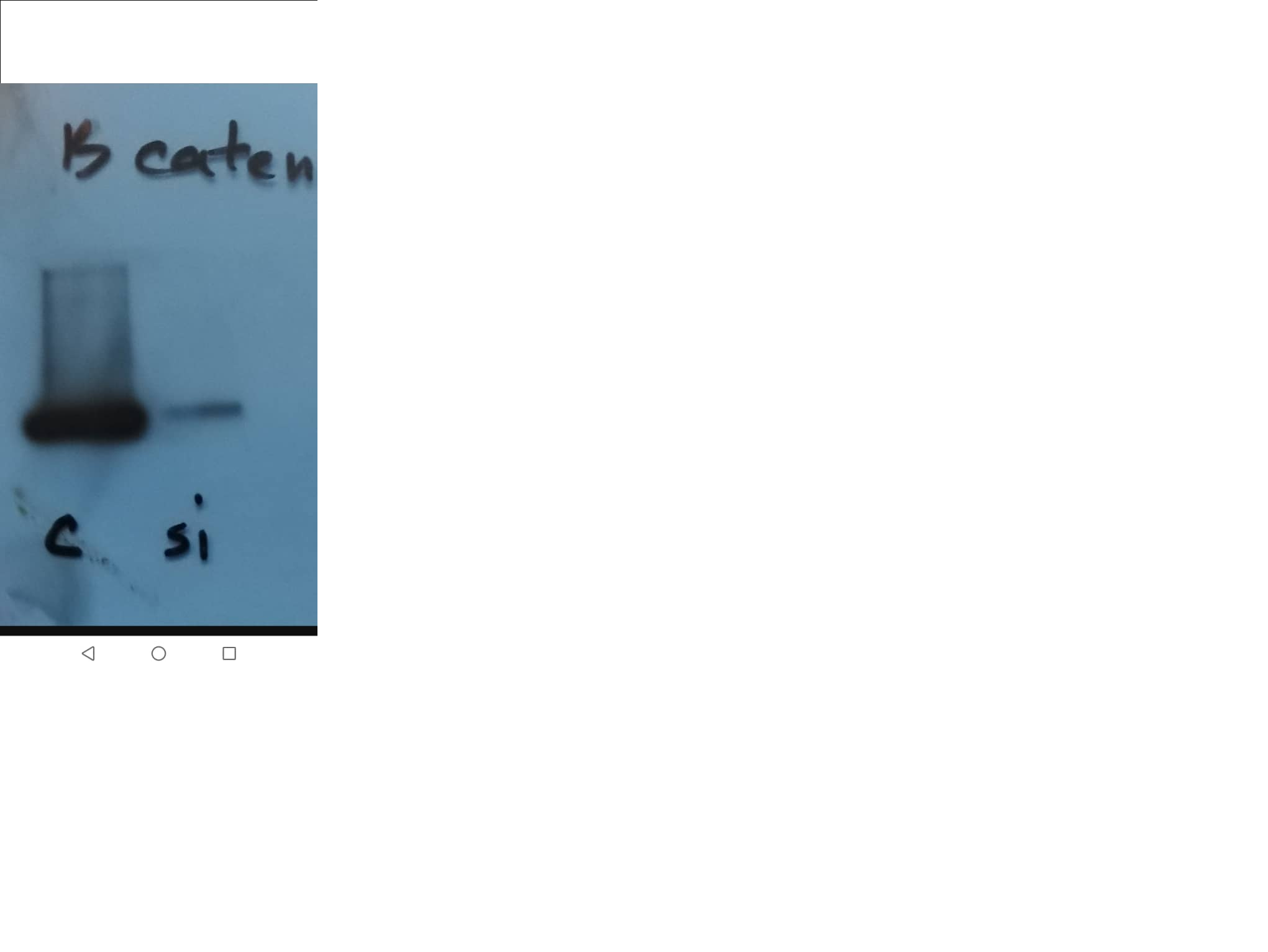

Supplement: Supplementary Materials — Uncropped Western blots corresponding to Figure 3(e). [file 8507373.f1.zip › ß-catenin.png]
